# Cross-species spill-over potential of the H9N2 bat influenza A virus

**DOI:** 10.1038/s41467-024-47635-4

**Published:** 2024-04-25

**Authors:** Rabeh El-Shesheny, John Franks, Ahmed Kandeil, Rebecca Badra, Jasmine Turner, Patrick Seiler, Bindumadhav M. Marathe, Trushar Jeevan, Lisa Kercher, Meng Hu, Yul Eum Sim, Kenrie P. Y. Hui, Michael C. W. Chan, Andrew J. Thompson, Pamela McKenzie, Elena A. Govorkova, Charles J. Russell, Peter Vogel, James C. Paulson, J. S. Malik Peiris, Robert G. Webster, Mohamed A. Ali, Ghazi Kayali, Richard J. Webby

**Affiliations:** 1 Center of Scientific Excellence for Influenza Virus, National Research Centre, Giza, Egypt; 2 Department of Infectious Diseases, St. Jude Children’s Research Hospital, Memphis, TN USA; 3 Human Link, Dubai, United Arab Emirates; 4 Department of Biology, Wanek School of Natural Science, High Point University, High Point, NC USA; 5 School of Public Health, The University of Hong Kong, Hong Kong, China; 6 Department of Immunology and Microbiology, The Scripps Research Institute, La Jolla, CA USA

**Keywords:** Influenza virus, Pathogens, Mouse, Viral transmission

## Abstract

In 2017, a novel influenza A virus (IAV) was isolated from an Egyptian fruit bat. In contrast to other bat influenza viruses, the virus was related to avian A(H9N2) viruses and was probably the result of a bird-to-bat transmission event. To determine the cross-species spill-over potential, we biologically characterize features of A/bat/Egypt/381OP/2017(H9N2). The virus has a pH inactivation profile and neuraminidase activity similar to those of human-adapted IAVs. Despite the virus having an avian virus–like preference for α2,3 sialic acid receptors, it is unable to replicate in male mallard ducks; however, it readily infects ex-vivo human respiratory cell cultures and replicates in the lungs of female mice. A/bat/Egypt/381OP/2017 replicates in the upper respiratory tract of experimentally-infected male ferrets featuring direct-contact and airborne transmission. These data suggest that the bat A(H9N2) virus has features associated with increased risk to humans without a shift to a preference for α2,6 sialic acid receptors.

## Abstract (web summary)

In this study, the authors report that a bat influenza A (H9N2) virus shows receptor binding features similar to avian influenza viruses, efficiently infects ex-vivo human respiratory cells and replicates in the lungs of mice and upper respiratory tract of ferrets following airborne transmission.

## Introduction

Emerging infectious diseases and pandemics in humans are often caused by pathogens transmitted from non-human animal reservoirs^1^. Influenza A viruses (IAVs) can be found in various animals, with occasional transmission between species^2^. Although waterfowl are the major natural reservoir for IAVs, infections in mammalian hosts pose the greatest threat to humans. Bats, accounting for approximately 20% of all global mammal species, carry RNA and DNA viruses asymptomatically and are natural reservoirs for multiple zoonotic viruses, including rabies, Nipah, Ebola, and coronaviruses^3–6^.

In 2009–2010, the first bat-associated IAV was discovered in an asymptomatic little yellow-shouldered bat (*Sturnira lilium*) in Guatemala. Although the virus had the key structural and genomic properties of IAVs, it was phylogenetically divergent from all known IAV subtypes. With its hemagglutinin (HA) and neuraminidase (NA) having only 45% and 24% amino acid identity, respectively, with those of other IAVs, the terminology HA-like and NA-like was adopted for these surface proteins^7^. This new virus (A/bat/Guat/2009) has, subsequently, been classified as a separate subtype A(H17N10)^8^. In 2010, another bat-associated influenza virus, A/bat/Peru/2010, was detected in asymptomatic New World flat-faced fruit bats (*Artibeus planirostris*) in Peru. This virus also could not be classified as any of the existing IAV subtypes, including A(H17N10), and was, therefore, classified as A(H18N11)^8^.

The HA and NA of the A(H17N10) and A(H18N11) viruses are functionally distinct from those of other IAVs^3^. Whereas conventional IAVs initiate virus replication by entering cells via their apical sides, bat A(H17N10) and A(H18N11) influenza viruses were shown to initiate infection of polarized MDCK II cells through the basolateral surface^9^. Unlike other IAV HAs, H17 and H18 do not use sialic acid as ligands for attachment. Instead, both H17 and H18 use the major histocompatibility complex class II molecule for entry into host cells^2,9^. The A(H18N11) virus replicated poorly in mice and ferrets but could infect Jamaican fruit bats, inducing minor signs of disease that were limited to nasal and ocular discharge with virus shedding in rectal specimens^10^. A(H17N10) and A(H18N11) bat viruses were initially detected in rectal swabs, which suggests a fecal–oral rather than a respiratory mode of transmission^11^.

The high A(H17N10) and A(H18N11) seroprevalence in the bat populations of Central America and South America, respectively, is evidence of the widespread geographic distribution and sustained transmission of these viruses in bat species^12^. In contrast, no evidence has been found of IAV in European bats^13^.

In 2017, a novel IAV (A/bat/Egypt/381OP/2017) was isolated in Egypt from Egyptian fruit bats (*Rousettus aegyptiacus*)^3,14^. This virus was phylogenetically distant from the A(H17N10) and A(H18N11) viruses and instead represented a distinct HA and NA lineage within the A(H9N2) subtype^3^. The remaining gene segments of the Egyptian virus also clustered phylogenetically with avian influenza viruses^3^. A/bat/Egypt/381OP/2017 was primarily detected in oral swabs and, unlike the previous bat influenza viruses, was successfully isolated in chicken eggs^3^. A study by Halwe et al. showed that inoculating Egyptian fruit bats with A/bat/Egypt/381OP/2017 led to a productive infection and seroconversion. Although viral RNA could not be detected in organs or swabs of contact animals, histopathologic analysis of the contact animals suggested that bat-to-bat transmission of the virus had indeed occurred^15^.

Together, the available data show that bats are hosts to at least two distinct forms of IAV. The first of these groups is represented by the A(H17N10) and A(H18N11) viruses, which are genetically and functionally separated from other IAVs. The genesis of these viruses is unclear. The second group is represented by the A(H9N2)-like virus, which is most parsimoniously explained by a bird-to-bat transmission event. The bat A(H9N2)-like virus has many amino acid changes when compared to avian A(H9N2) viruses, yet the impact of these changes with regard to human health risk is unknown.

In this study, we extensively characterize the A/bat/Egypt/381OP/2017 virus to identify the capacity of bats to act as an intermediate host selecting for viral traits associated with mammalian influenza viruses. Our results show that, although the bat A(H9N2)-like virus binds preferentially and strongly to α2,3 glycans, a property associated with avian influenza viruses, it is capable of infecting ex vivo human respiratory cell cultures and replicates in the lungs of mice and in the upper respiratory tract of ferrets, properties associated with human influenza viruses.

## Results

### HA activation and inactivation pH of the Egyptian bat virus

**Fig. 1:**
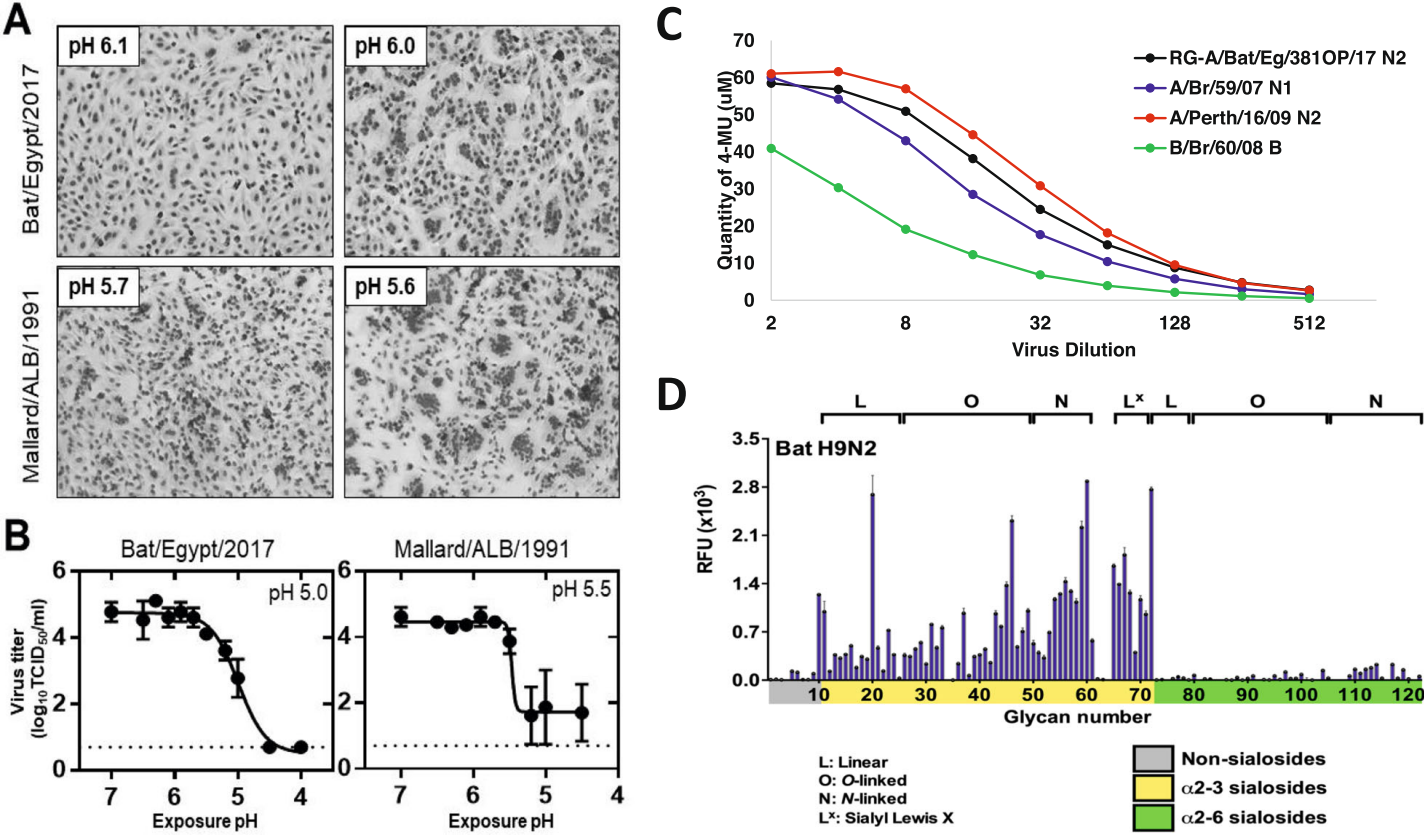
Phenotypic properties of A/bat/Egypt/381-OP/2017 (H9N2) influenza virus. **A** Syncytium formation assay. Monolayers of Vero cells were infected with viruses at an MOI of 3 PFU/cell. At 16 h post infection, cells were treated with TPCK-trypsin, washed, and then treated with pH-adjusted buffers. Cells were then left to recover for 3 h, washed, fixed, and stained for microscopy. Representative images from three independent experiments are shown. **B** Acid inactivation assay. Viruses were exposed to pH-adjusted buffers at 37 °C for 1 h then neutralized, after which the infectious virus titer was measured by TCID_50_ assay in MDCK cells. Data from three independent experiments were analyzed with a non-linear regression model by GraphPad Prism, and the calculated virus inactivation pH_50_ values are shown. **C** NA activity of human influenza A and B viruses and bat influenza virus as measured by a modified fluorescence-based assay. **D** Receptor specificity of A/bat/Egypt/381-OP/2017 (H9N2) by glycan microarray. Binding results are presented as bar graphs with bars representing the averaged mean signal derived from six individual replicates of each glycan, with highest and lowest signals removed to give a final average of four median replicates. Error bars represent standard error of the averaged signal. Source data are provided as a Source data file.

HA stability, or the pH at which the HA protein becomes activated for membrane fusion or inactivated in the absence of target cells, contributes to IAV pathogenicity and transmissibility^16^. To measure the HA acid stability of A/bat/Egypt/381-OP/2017 and A/mallard/Alberta/17/1991, we conducted syncytium formation and virus inactivation assays (Fig. 1A, B). For A/mallard/Alberta/17/1991, the highest pH value at which the HA protein was activated to induce syncytium formation was 5.6, and the midpoint of pH-induced inactivation of infectivity was pH 5.5. In contrast, A/bat/Egypt/381-OP/2017 had an HA activation pH of 6.0 and an inactivation pH of 5.0 (with a 90% reduction in titer at pH 5.3). Such disparities in HA activation pH versus virus inactivation pH have been observed for several swine H1N1 and H3N2 viruses^17^. At the midpoint of inactivation of A/mallard/Alberta/17/1991 (pH 5.5), the titer of A/bat/Egypt/381-OP/2017 was reduced by only 0.5 log_10_ when compared to the titer at higher pH values (Fig. 1B). Therefore, A/bat/Egypt/381-OP/2017 shows resistance to inactivation by exposure to extracellular pH that is similar to that of human-adapted IAV^16^.

### Neuraminidase activity assay

NA subtypes of IAV can be divided into two groups: group 1, which includes N1, N4, N5, and N8, and group 2, which includes N2, N3, N6, N7, and N9^18^. The N10 bat NA, which is highly divergent from the well-established N1–N9 subtypes of IAV NA, lacks NA activity^19^. To determine whether A/bat/Egypt/381-OP/2017 possessed sialidase activity, we used a modified fluorescence-based assay to measure the NA activity of the virus and of control human influenza A and B viruses. The NA activity of A/bat/Egypt/381-OP/2017 was comparable to that of A/Brisbane/59/2007 (H1N1) (which has a group 1 NA) and A/Perth/16/2009 (H3N2) (which has a group 2 NA) (Fig. 1C), indicating that the A(H9N2)-like virus had maintained sialidase activity upon propagation in bats.

### Receptor specificity

We had previously shown that A/bat/Egypt/381-OP/2017 had receptor-binding residues typical of most avian influenza viruses, with a receptor preference for α2,3-linked sialic acids, as measured by solid-phase binding^3^. To extend these studies, we conducted a glycan microarray analysis to examine the receptor specificity of A/bat/Egypt/381-OP/2017 in finer detail. The glycan microarray comprised synthetic glycans representing various natural sialylated O-linked and N-linked glycans, of which 62 were capped with α2,3-linked sialic acids (avian type) and 50 with α2,6-linked sialic acids (human type). We found that the A/bat/Egypt/381-OP/2017 (H9N2) virus bound exclusively to avian type α2,3-sialosides, including O-linked glycans, N-linked glycans, and linear fragments, including structures terminating with the sialy-Lewis X determinant (NeuAcα2-3Galβ1-4[Fucα1-3]GlcNAc) (Fig. 1D and Supplementary Table 1). The only structures with α2,3-linked sialic acids that were not bound were those that had sialic acid as a branch to an internal galactose (glycans 62–64). Therefore, this bat virus exhibits a receptor specificity typical of avian H9N2 viruses isolated in North America^20^.

### Ex vivo replication efficiency in the human respiratory system

**Fig. 2:**
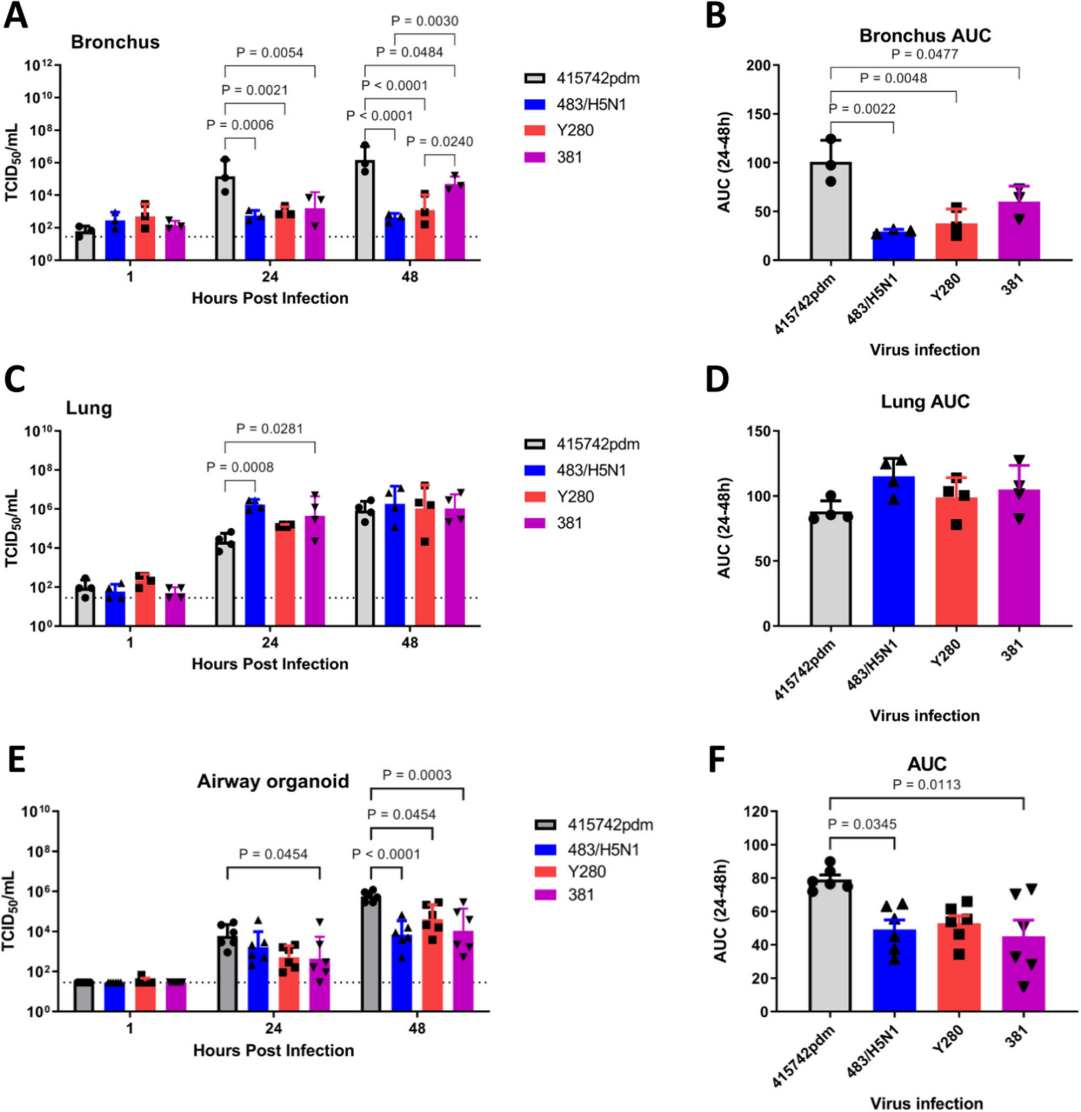
Replication of avian, bat, and human-origin influenza viruses in ex vivo cultures of human bronchus and lung cells and in human airway organoids. **A**, **C** Human bronchial (*n* = 3 individual donors) and lung tissues (*n* = 4 individual donors) were infected with A/Hong Kong/415742/2009 (415742pdm), A/Hong Kong/483/1997 (483/H5N1), A/Duck/Hong Kong/Y280/97 (Y280), or A/bat/Egypt/381-OP/2017 (381) at 1 × 10^6^ pfu/mL at 37 °C. Viral titers in culture supernatants collected at 1, 24, and 48 h after infection were determined by TCID_50_ assays in MDCK cells. **B**, **D** The viral loads from (**A**, **C**) are depicted as areas-under-the-curve (AUCs). **E** Human airway organoids (*n* = 6 individual donors) were infected with the above viruses at 1 × 10^6^ pfu/mL at 37 °C. Viral titers in culture supernatants collected at 1, 24, and 48 h after infection were determined by TCID_50_ assays in MDCK cells. **F** Viral titers from (**E**) are depicted as AUCs. Bar charts show the data as the mean + SD of the results for at least three individual donors. The horizontal line denotes the limit of detection in the TCID_50_ assay. Statistical analysis was performed using two-way ANOVA with Tukey’s post-test (**A**, **C**, **E**) or one-way ANOVA followed by Tukey’s post-test (**B**, **D**, **F**). *P* < 0.05 was considered to indicate statistical significance, and exact *P* values are presented. Source data are provided as a Source data file.

To model the replication potential of A/Bat/Egypt/390OP/2017 in humans, bronchus and lung cells were grown in culture at an air–liquid interface and infected with A/Bat/Egypt/381 OP/2017 (H9N2), A/Hong Kong/483/1997 (H5N1) and A/Duck/Hong Kong/Y280/97 (H9N2) as representative avian influenza viruses, and A/Hong Kong/415742/2009 (H1N1pdm09) as a representative human virus. Consistent with its human origin, A/Hong Kong/415742/2009 replicated to significantly higher titers (*P* < 0.05) than any other virus in bronchus cells (Fig. 2A, B), whereas the viruses of bat origin replicated at low levels in bronchus cells, similar to the levels observed with the avian viruses. In contrast to their low replication in bronchus cells, the bat and avian viruses replicated to high titers in lung cells, attaining titers similar to those of the human virus and consistent with the higher concentration of α2,3-sialosides in lung tissue (Fig. 2C, D). Although all tested viruses, including bat-origin H9N2 viruses, replicated in airway organoids, the human virus had the highest viral titers and areas-under-the-curve (AUC) among the viruses compared.

### Replication in alveolar epithelial cells

**Fig. 3:**
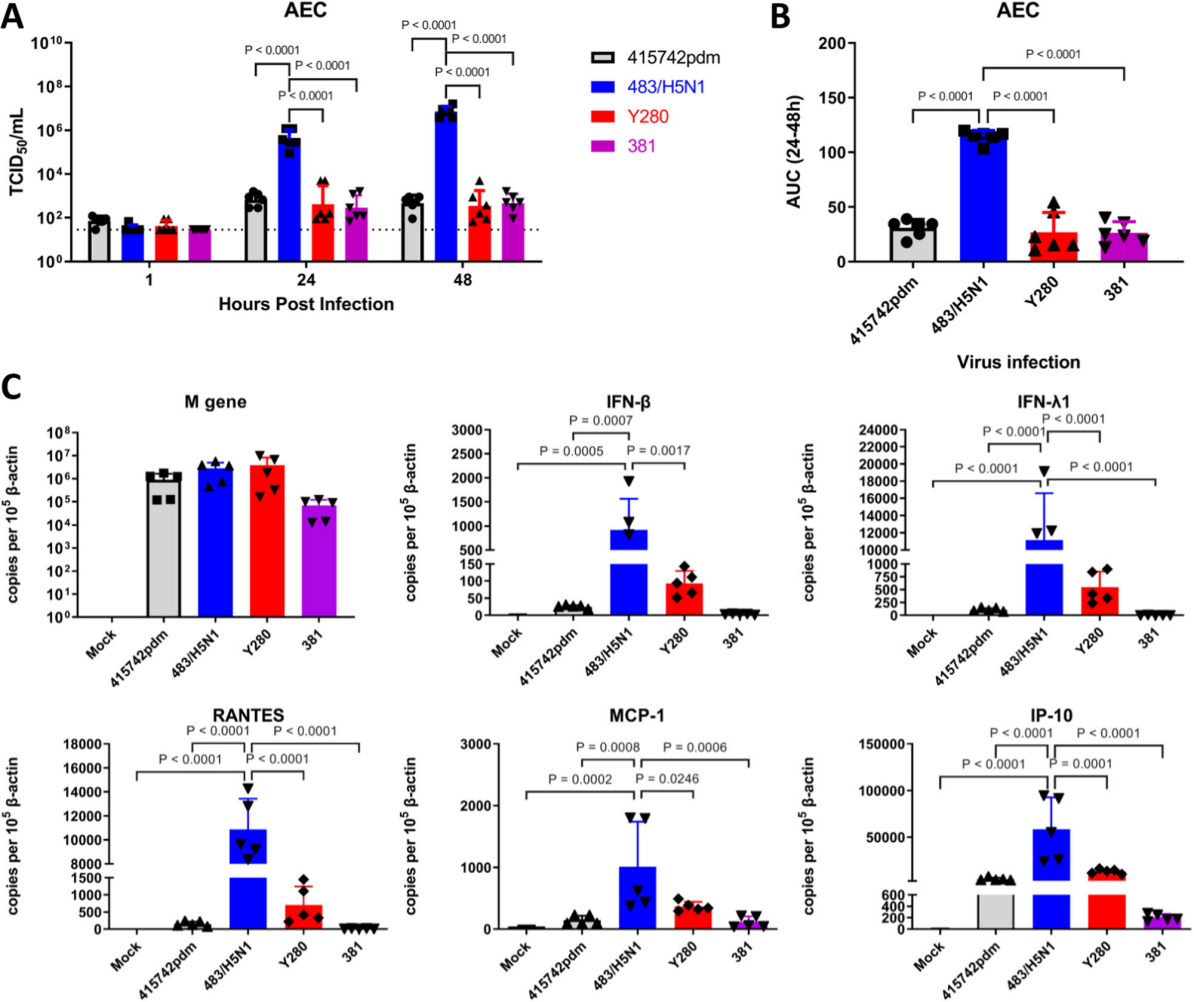
Virus titers and cytokine and chemokine gene expression detected in the supernatant of infected alveolar epithelial cells. **A** Alveolar epithelial cells (AECs) were infected with the A/Hong Kong/415742/2009 (415742pdm), A/Hong Kong/483/1997 (483/H5N1), A/Duck/Hong Kong/Y280/97 H9N2 (Y280), or A/bat/Egypt/381-OP/2017 H9N2 (381) viruses at an MOI of 0.01 and maintained in culture at 37 °C. Viral titers in culture supernatants collected at 1, 24, and 48 h post inoculation (hpi) were determined by TCID_50_ assays in MDCK cells. Bar charts show the data as the mean +/− SD (*n* = 6 individual donors). **B** Viral titers from panel A are depicted as AUCs. Bar charts show the data as the mean + SD (*n* = 6 individual donors). The horizontal line denotes the limit of detection of the TCID_50_ assay. **C** AECs were infected with the indicated viruses at an MOI of 2 and maintained in culture at 37 °C. Expression of the mRNA of viral M genes and of the mRNAs encoding cytokines (IFN-β and IFN-λ1) and chemokines (IP-10; regulated on activation, normal T cell–expressed and secreted [RANTES]; and MCP-1) in AECs at 24 hpi is shown. Bar charts show the data as the mean + SD of the results for five individual donors. Statistical analysis was performed using two-way ANOVA with Tukey’s post-test (**A**) or one-way ANOVA followed by Tukey’s post-test (**B**, **C**); *P* < 0.05 was considered to indicate statistical significance, and exact *P* values are presented. Source data are provided as a Source data file.

We next assessed the replication and subsequent cytokine expression of the bat and control viruses in cultured primary human alveolar epithelial cells (AECs). Except for A/Hong Kong/483/1997 (H5N1), no significant differences in titers were observed among tested viruses when infecting AECs (Fig. 3). To assess the expression of cytokines (IFN-β and IFN-λ1) and chemokines (IP-10, RANTES, and MCP-1), RNA was extracted from infected AECs and the mRNAs encoding cytokines and chemokines were quantified. A/Hong Kong/483/1997 (H5N1) induced the highest levels of mRNA for all tested cytokines and chemokines, whereas A/Bat/Egypt/381-OP/2017 showed a trend to induce the least mRNA encoding cytokines and chemokines.

### Pathogenicity of A/bat/Egypt/381-OP/2017 in mice

**Fig. 4:**
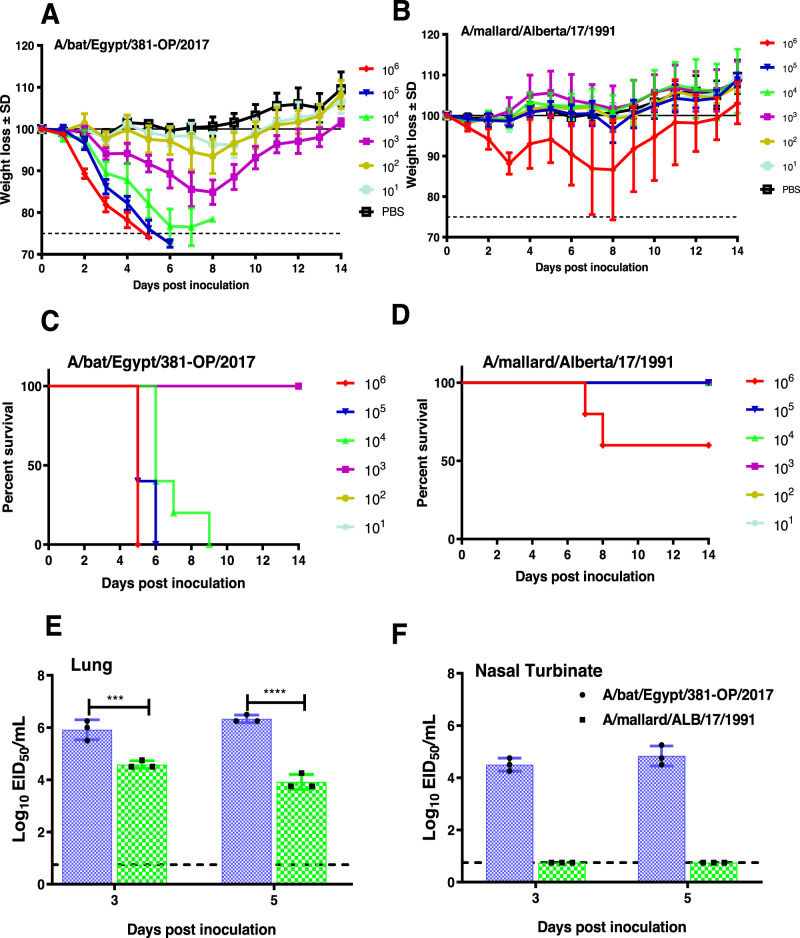
Pathogenicity of A/bat/Egypt/381-OP/2017 (H9N2) and A/mallard/Alberta/17/1991 (H9N2) viruses in mice. Groups of 5- to 6-week-old DBA/2J mice (*n* = 5) were inoculated i.n. with the indicated doses (10^1^, 10^2^, 10^3^, 10^4^, 10^5^, or 10^6^ EID_50_) of A/bat/Egypt/381-OP/2017 (H9N2) and A/mallard/Alberta/17/1991 (H9N2) viruses. The mean values +/− SD of body weight loss (**A**, **B**) and survival (**C**, **D**) were evaluated daily for 14 days. Groups of mice (*n* = 3) that were infected with 10^6^ EID_50_ were euthanized at 3 or 5 dpi (**E**, **F**), and their lungs and nasal turbinates were harvested, homogenized, and used to quantify the viral titers by EID_50_ assays. Viral titers expressed as the log_10_ EID_50_/ml were plotted as the mean. Statistical analysis was performed using two-way ANOVA (***P < 0.001, ****P < 0.0001). Source data are provided as a Source data file.

To evaluate the pathogenicity of A/bat/Egypt/381-OP/2017 in mice, we inoculated DBA/2J animals with A/bat/Egypt/381-OP/2017 and A/mallard/Alberta/17/1991 A(H9N2) viruses. A/bat/Egypt/381-OP/2017 was substantially more virulent than A/mallard/Alberta/17/1991 (Fig. 4A, B). A/bat/Egypt/381-OP/2017 induced weight loss of up to 15% of starting body weight at a dose of 10^3^ EID_50_, whereas A/mallard/Alberta/17/1991 induced weight loss only at a dose of 10^6^ EID_50_ (Fig. 4C, D). A/bat/Egypt/381-OP/2017 infected mice met human endpoints at doses of 1 × 10^4^ EID_50_ and above. Consistent with the differences in morbidity and mortality, significantly higher viral loads were detected in the lungs of mice infected with A/bat/Egypt/381-OP/2017 than in the lungs of mice infected with A/mallard/Alberta/17/1991 (Fig. 4E). Only A/bat/Egypt/381-OP/2017 was detected in nasal turbinates (Fig. 4F).

To further detail the pathologic consequences of virus infection in mice, we immunostained fixed tissue sections for viral nucleoprotein antigen. At 3 days post inoculation (dpi), A/bat/Egypt/381-OP/2017 infection was characterized by diffuse infection of respiratory epithelial cells in the airways of the nose and lungs. At the same time, there was extremely limited infection of and damage to the alveolar epithelium (Supplementary Figs. 1A–C, G–I and 2A, B). By 5 dpi, there was extensive loss of respiratory epithelium in the nose, trachea, and bronchioles of mice infected with A/bat/Egypt/381-OP/2017 (Supplementary Figs. 1M–O, S–U and 2E). In marked contrast to the extensive A/bat/Egypt/381-OP/2017 infection by 3 dpi, animals inoculated with A/mallard/Alberta/17/1991 showed no detectable infection of the nasal respiratory epithelium and just a few patches of infected respiratory epithelium in the trachea and bronchioles (Supplementary Figs. 1 and 2). There were still no A/mallard/Alberta/17/1991-infected cells or lesions in the nose on day 5 post infection, but virus-infected cells were diffusely scattered within well-defined areas of the lungs in two of the three mice.

### Pathogenicity and transmission of A/bat/Egypt/381-OP/2017 in ferrets

**Fig. 5:**
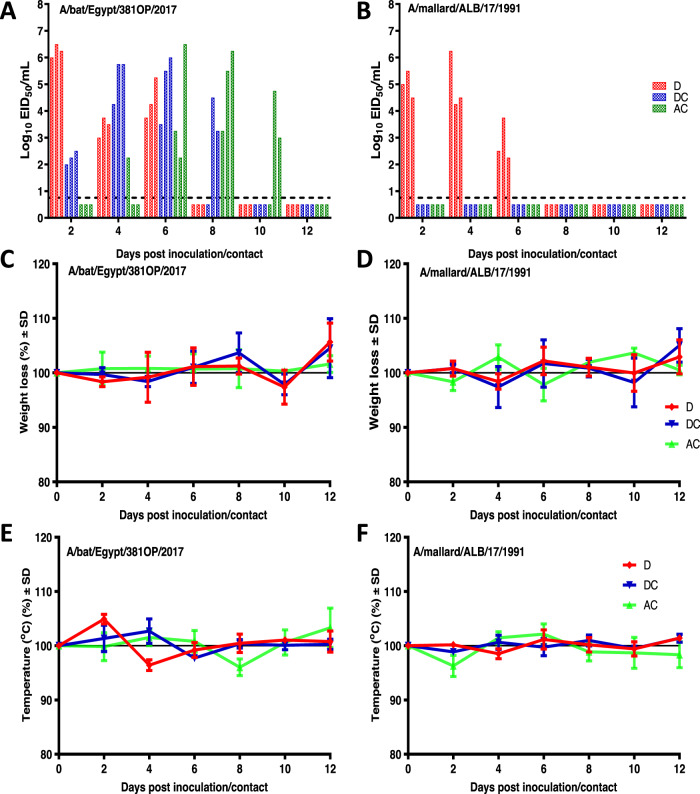
Pathogenicity and replication of A/bat/Egypt/381-OP/2017 and A/mallard/Alberta/17/1991 viruses in ferrets. **A**, **B** Ferrets (*n* = 3/group) were inoculated i.n. with 10^6^ EID_50_ of each virus studied. Each ferret was paired with an individual naïve ferret at 24 h post inoculation. Viral titers in nasal washes from individual inoculated ferrets (D; red), direct-contact ferrets (DC; blue), and airborne-contact ferrets (AC; green) on the days post inoculation or post contact were determined and presented as the log_10_EID_50_/mL. The mean values +/− SD of body weight loss (**C**, **D**) and changes in body temperature (**E**, **F**) of individually inoculated (D), direct-contact (DC), and airborne-contact (AC) ferrets up to 12 days post inoculation or post contact and the data are presented as mean values +/− SD using GraphPad Prism. Source data are provided as a Source data file.

To further assess the zoonotic risk of A/bat/Egypt/381-OP/2017, we conducted ferret transmission studies. Ferrets inoculated with A/bat/Egypt/381-OP/2017 or A/mallard/Alberta/17/1991 attained peak mean titers in nasal washes of 6.25 ± 0.25 and 5 ± 0.5 log_10_EID_50_/mL, respectively, at 2 dpi (Fig. 5A, B). Clinical signs in the infected animals were mild with both viruses (Fig. 5C–F); the only detectable change was a slight increase in temperature at 2 dpi in each of the animals infected with A/bat/Egypt/381-OP/2017 (Fig. 5C). A/bat/Egypt/381-OP/2017, but not A/mallard/Alberta/17/1991, transmitted to contact ferrets by 2 dpc, as demonstrated by the detection of virus in nasal secretions (Fig. 5B, C) and seroconversion at 14 days post contact (Supplementary Table 4). Similarly, A/bat/Egypt/381-OP/2017 was detected in the nasal washes of all airborne-contact animals, whereas no virus was detected in contacts of A/mallard/Alberta/17/1991 (Fig. 5A, B). Virologically confirmed airborne transmission was further confirmed by demonstration of seroconversion (Supplementary Table 4). Of note, the infections with A/bat/Egypt/381-OP/2017 in airborne-contact animals were accompanied by a slight decrease in body temperature around day 9 or 10, concomitant with virus detection in nasal washes.

**Fig. 6:**
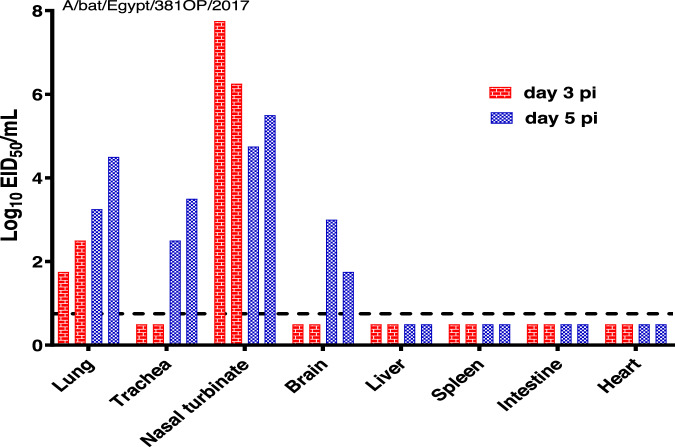
Replication of A/bat/Egypt/381-OP/2017 in ferret tissues. Ferrets (*n* = 2) were inoculated with 10^6^ EID_50_ of virus and then humanely euthanized on days 3 and 5 post inoculation. Tissues were collected, and infectious viral titers were determined by EID_50_ assays. Each bar represents an individual ferret, and the dotted line indicates the lower limit of virus detection by the assay (1.0 log_10_EID_50_/mL). Source data are provided as a Source data file.

To examine the virus distribution in animals, ferrets (*N* = 2 at each timepoint) infected with A/bat/Egypt/381-OP/2017 were euthanized at 3 and 5 dpi and their lung, trachea, nasal turbinate, brain, liver, spleen, intestine, and heart tissues were collected for virus titration (Fig. 6). At 3 dpi, virus was detected in lung and nasal turbinate tissue. The infected tissue expanded by 5 dpi to include the trachea and brain, albeit with only moderate to low levels of virus being present in those organs.

To confirm some of the tissue distribution findings, we immunostained 3 dpi tissue for influenza virus nucleoprotein. In the respiratory tract, scant virus antigen was detected in the lungs, consistent with the low infectious titers detected (Supplementary Fig. 3). There was extensive sloughing of olfactory neuroepithelium; however, there was only multifocal involvement of respiratory epithelium in the nose and the nasopharynx was spared. There was diffuse labeling of affected epithelium, which was characterized by anoikis. Minimal extension into olfactory nerves was detected.

### Replication and transmission in mallard ducks

To assess the ability of A/bat/Egypt/381-OP/2017 and A/mallard/Alberta/17/1991 to replicate in mallard ducks, we infected birds at a dose of 10^6^ EID_50_. No infected ducks exhibited clinical symptoms during the 14-day observation period. A/bat/Egypt/381-OP/2017 appeared to be unable to replicate in mallard ducks, with no infectious virus or seroconversion being detected in infected or contact birds (Supplementary Fig. 4B, D and Supplementary Table 5). Conversely, A/mallard/Alberta/17/1991 was detected in cloacal swabs of infected and contact ducks (10^0.75^–10^7.75^ EID_50_/mL); virus was detected only sporadically in oropharyngeal swabs (Supplementary Fig. 4A, C and Supplementary Table 5).

## Discussion

The identification of the A(H17N10) and A(H18N11) viruses that are genotypically and phenotypically distinct from other influenza A viruses suggests that bats harbor their own lineages of virus. Although the origin of these viruses is unclear, the phylogenetic positioning of their internal genes at the base of influenza A genes, just downstream of the influenza A/B split^1^, could mean that the viruses have been in bats for a long time. Without definitive precursors, it has been difficult to assess the impact of bat adaptation of an influenza virus on the risk posed to humans. However, the identification of the A(H9N2)-like virus offers the possibility of investigating this impact. This virus has been in bats for a shorter period than the A(H17N10) and A(H18N11) viruses and is clearly derived from avian A(H9N2) viruses. The objective of this study was to examine the phenotypic properties of the bat A(H9N2) virus and compare it with their avian-origin counterparts.

Avian influenza viruses generally exhibit a strong preference for α2,3-sialoside receptors. However, this is not always the case for A(H9N2) viruses. In the 1990s, A(H9N2) viruses became established in land-based poultry, and over time a significant number of isolates have been identified with Leu 226 in the hemagglutinin, which is considered a signature for human-type receptor specificity^20–22^. Indeed, A(H9N2) viruses with Leu 226 were found to bind human-type α2,6-sialoside receptors, and this mutation increases infection and contact transmission in ferrets, raising concern about pandemic potential^21,23,24^. In this regard, it is of interest that the bat A(H9N2) viruses have Gln at 226, which is a signature of avian virus receptor specificity. Consistent with previous findings^3^ and in contrast to the finding that A(H17N10) and A(H18N11) viruses have evolved to use entirely different receptors^2,10^, we found that A/bat/Egypt/381-OP/2017 bound preferentially and strongly to α2,3 glycans. The binding to α2,3-sialosides is consistent with findings that little brown bats (*Myotis lucifugus*), which are widely distributed in North America, contain both α2,3 and α2,6 sialic acids throughout their respiratory tracts^25^. Assuming similar distributions in other bat species, this would suggest that there is little selective pressure for avian influenza viruses to evolve α2,6 binding upon bat adaptation. The bat A(H9N2) virus also maintained typical influenza A virus NA function, a feature that has been lost in the A(H17N10) and A(H18N11) viruses. Consistent with the avian-like molecular signatures of the bat A(H9N2) viruses, their replication in primary cells of human origin more closely resembled that of the control avian viruses than that of the control human virus. This was manifested as lower replication in bronchus and airway organoids but similar replication in lung cell cultures when compared with the human A(H1N1)pdm09 virus. It is not clear why the bat virus replicated better in lung culture compared to airway organoid culture requiring further studies.

Somewhat paradoxically, and despite receptor binding features that were most similar to those of avian influenza viruses, the bat A(H9N2) virus had other properties that were more similar to those of mammalian-adapted viruses. The pH stability of the HA is an important component of avian influenza adaptation to mammalian hosts^26^. A/bat/Egypt/381-OP/2017 had activation and inactivation pH values of 6.0 and 5.0, respectively. An activation pH of 6.0 is a more characteristic of an avian virus, whereas an inactivation pH of 5.0 is more characteristic of a human virus^19^. Although human viruses typically have similar activation and inactivation pH values, some swine viruses have been found to have mismatches. The ability of A/bat/Egypt/381-OP/2017 to transmit between ferrets, and although requiring further examination, suggests that HA inactivation is a better predictor of transmission potential than is HA activation in ferret models. It was this ability to transmit via contact and airborne routes that was the most striking and unexpected feature of A/bat/Egypt/381-OP/2017, especially considering its receptor usage. Direct and, more rarely, airborne transmission of A(H9N2) viruses have been reported, but typically in the setting of viruses that were isolated from humans^27^ or that possessed some level of α2,6-sialoside binding capacity^21,28^. The robust transmission of A/bat/Egypt/381-OP/2017 to both sets of contact animals does imply an elevated risk to human health. The ability to transmit between ferrets is a highly weighted element in the World Health Organization’s Tool for Influenza Pandemic Risk Assessment (TIPRA) (https://www.who.int/publications/i/item/tool-for-influenza-pandemic-risk-assessment-(tipra)-2nd-edition) and the Influenza Risk Assessment Tool (IRAT) of the US Centers for Disease Control and Prevention^29^. TIPRA and IRAT are designed to assign the relative pandemic risk of animal influenza viruses, using scoring elements, including ferret transmission, selected by subject matter experts. Nonetheless, it is worthwhile repeating ferret transmission studies using other experimental designs. Although the bat A(H9N2) virus could propagate in eggs, it was unable to replicate to any detectable level in mallard ducks, the natural reservoir of IAV. An inability to replicate in ducks has also been reported for poultry-adapted A(H9N2) viruses^30^, and it is unclear whether this is necessarily a consequence of replication in bats.

Our data indicate that the bat A(H9N2)-like virus has a mix of biologic properties, some typically associated with avian influenza viruses, some with human influenza viruses. This mix of phenotypes makes it difficult to assess the pandemic risk of the virus in relation to other zoonotic threats. The capacity of the virus to transmit between ferrets and its HA stability profile do, however, indicate a certain level of zoonotic threat. Therefore, further investigating the abundance and nature of influenza viruses in bats and comparisons with other avian viruses with different levels of mammalian adaptations appears prudent.

## Methods

### Ethics statement and facility

The animal studies were approved by the Animal Care and Use Committee of St. Jude Children’s Research Hospital (IACUC) (protocol number 428) and were performed according to the guidelines set by the committee. All experiments with infectious A(H9N2) viruses were conducted in an Animal Biosafety Level 3 enhanced facility.

### Cells and viruses

Madin–Darby canine kidney (MDCK) cells (ATCC CCL-34) and African green monkey kidney (Vero) cells (ATCC CCL-81) were obtained from the American Type Culture Collection (ATCC) (Manassas, VA) and were maintained in MEM (cellgro; Corning, Manassas, VA) supplemented with 5% (for MDCK cells) or 10% (for Vero cells) fetal calf serum (HyClone, Logan, UT) and a mixture of antibiotics and antimycotics (100 U of penicillin, 0.1 mg of streptomycin, and 0.25 μg of amphotericin B per milliliter) (Gibco).

A/mallard/Alberta/17/1991 (H9N2) was selected as a control virus as it was an ancestral avian virus with 99% protein sequence similarity to the bat virus and was available in our collection. A/bat/Egypt/381-OP/2017 (H9N2) and A/mallard/Alberta/17/1991 (H9N2) viruses were propagated and titrated by 50% egg infectious doses (EID_50_) in the allantoic cavities of 10-day-old embryonated chicken eggs at 35 °C for 48 h. Allantoic fluid was pooled from multiple eggs, clarified by centrifugation, and frozen in aliquots at −80 °C. Virus titers were determined by injecting 100 μL of serial 10-fold dilutions of virus into the allantoic cavities of 10-day-old embryonated chicken eggs (eggs) and then calculating the EID_50_ by the method of Reed and Muench^31^. A highly pathogenic avian influenza (HPAI) A(H5N1) virus (A/Hong Kong/483/1997, 483/H5N1) isolated from a fatal human infection, a 2009 pandemic influenza virus (A/Hong Kong/415742/2009, 415742pdm) isolated from a patient in Hong Kong, and duck A(H9N2) viruses (A/duck/Hong Kong/Y280/97, Y280) were used as controls for ex vivo replication efficiency studies in human respiratory system.

Furthermore, HA and NA segments of A/bat/Egypt/381-OP/2017 (H9N2) virus were cloned into dual-promoter expression vector pHW2000. Plasmids encoding cDNAs of HA and NA segments of A/bat/Egypt/381-OP/2017 (H9N2) and six plasmids encoding the remaining segments of A/Puerto Rico/8/1934 (H1N1) virus were transfected into HEK293T cells (ATCC CRL-3519) by using Lipofectamine 3000 reagent (Thermo Fisher). At 48 h post transfection, the cell supernatant was harvested, and 0.2 mL of the supernatant was injected into 10-day-old embryonated chicken eggs to propagate the virus. The rg virus was used in HA stability assay, glycan array, and neuraminidase activity assay.

### HA stability assay

The HA activation pH was measured by a syncytium formation assay^32^. In brief, monolayers of Vero cells were infected with viruses at a multiplicity of infection (MOI) of 3 plaque-forming units (PFU) per cell for 1 h at 37 °C. At 16 h post inoculation (hpi), the cells were incubated in TPCK-treated trypsin for 10 min, washed, and incubated with pH-adjusted DPBS buffers for 10 min before removal. The cells were then neutralized by adding DMEM with 5% FBS and incubated at 37 °C for 3 h. They were then washed with PBS, fixed with methanol, and stained with a Hema 3 Stat Pack staining kit (Fisher Scientific, Rochester, NY), used according to the manufacturer’s instructions. Representative images were acquired with a Nikon D70 digital camera attached to a Nikon Eclipse TS100 inverted microscope. To measure the virus inactivation pH, aliquots of virus were incubated in pH-adjusted PBS buffers for 1 h at 37 °C, after which they were neutralized and the infectious virus titer was determined by tissue culture infectious dose (TCID_50_) assay in MDCK cells^33^. The curves were fitted to a nonlinear regression model, and the values for virus inactivation at pH 5.0 were calculated using GraphPad Prism software.

### Neuraminidase activity assay

Neuraminidase activity was measured by a fluorometric assay using 2′-(4-methylumbelliferyl)-α-D-*N*-acetylneuraminic acid (MUNANA) (Sigma-Aldrich) as a substrate^34^. All tested viruses were standardized to an equivalent dose of 10^7.0^ TCID_50_/mL, and two-fold serial dilutions of the virus were incubated with MUNANA at a final concentration of 100 μM for 30 min at 37 °C in 96-well flat-bottom, black, opaque plates (Corning Costar, Corning, NY). The reaction was terminated by adding a stop solution of 25% ethanol and 12.5% glycine (Fisher Scientific, Rochester, NY) in distilled water. Two-fold serial dilutions of 4-methylumbelliferone (4-MU) were used for each assay plate, with a concentration range of 0.6–80 µM to generate a standard curve. The fluorescence of the released 4-MU was measured with a Synergy 2 multimode microplate reader (BioTek Instruments, Winooski, VT), using excitation and emission wavelengths of 360 and 460 nm, respectively. The results were calculated as the means from two or three independent determinations and were expressed as the amount of released 4-MU in micromoles.

### Glycan binding

A custom glycan binding array, consisting of 122 glycans, was used^35^. In brief, viruses analyzed on glycan binding arrays were inactivated with 0.1% (vol/vol) β-propriolactone. Inactivated virus stocks with a titer of 2056 hemagglutination units (HAU/ml) were diluted to a final titer of 512 HAU/ml in PBS containing 1% BSA and incubated on the array surface in a humidified chamber protected from light for 1 h at room temperature. The virus was then removed, and the array was washed three times with PBS, pH 7.4. For detection, virus-incubated arrays were overlaid with anti-H9N2 polyclonal ferret antiserum (diluted 1:10 in PBS containing 1% BSA) for 1 h at room temperature. The antiserum was then removed, and the array was washed a further three times with PBS, pH 7.4. Finally, antiserum-labeled virus was detected with Goat anti-Ferret IgG-FITC (Fisher Scientific; diluted 1:100 in PBS containing 1% BSA). Arrays were then dipped three times in PBS and then three times in distilled water. The slides were dried by centrifugation and scanned using a ProScanArray Express HT confocal slide scanner (PerkinElmer). Signal data were collected using ImaGene software (BioDiscovery), and the signal data were processed to determine the average values (mean signal minus mean background) for four replicate spots on the array for each unique printed glycan. A complete summary of the array experiment and raw data in MIRAGE format^36^ are provided in Supplementary Tables 1–3.

### Ex vivo cultures and infection of the human respiratory tract

Fresh non-tumor bronchus and lung tissues were obtained from patients undergoing elective surgery in the Department of Surgery at Queen Mary Hospital, Hong Kong. The use of human respiratory tract tissue (lung and bronchus) to setup ex vivo explant culture for studying influenza and coronavirus infection was granted by the Institutional Review Board of the University of Hong Kong and the Hospital Authority (Hong Kong West) (approval no. UW 20-862). Consent forms were signed by participants. All consent forms were collected and kept by the operating surgeon.

The tissues were removed as part of clinical care, but the surplus was used for routine diagnostic requirements as detailed previously^37^. Tissues collected were convenience samples. The fragments of human tissues were infected with each virus at 1 × 10^6^ pfu/mL for 1 h at 37 °C. Mock-infected tissues served as negative controls. The explants were washed three times with PBS and placed in culture medium (F-12K nutrient mixture with L-glutamine and antibiotics), with or without a sterile surgical pathology sponge to establish an air–liquid interface condition, in 24-well culture plates, which were incubated at 37 °C in 5% CO_2_. Infectious viral titers in the culture supernatants were assessed at 1, 24, and 48 hpi by titration in MDCK cells. At 48 hpi, bronchus and lung tissues were fixed in 10% formalin and processed for immunohistochemical staining.

### Influenza virus infection of human airway organoids

Three-dimensional (3D) human airway organoids were generated from adult stem cells isolated from the non-tumor lung tissue obtained from patients undergoing lung resection in the Department of Surgery at Queen Mary Hospital. Organoid cultures (approximately 100 µm in diameter) were extracted from droplets of Matrigel (Growth Factor Reduced Basement Membrane Matrix; Corning) by using Gentle Cell Dissociation Reagent (STEMCELL Technologies) and sheared by mechanical disruption with 1-mL pipettes to allow viruses to gain access to the apical and basolateral sides of the epithelium. Around 100–200 organoids were infected with each influenza virus at 10^6^ pfu/mL for 1 h at 37 °C. Organoids were washed with culture medium three times, re-embedded in Matrigel, and plated in prewarmed 24-well suspension culture plates (Greiner). Once solidified, the Matrigel droplets were maintained in complete organoid medium^38^, and incubated at 37 °C in 5% CO_2_. The viral titers in the culture supernatants were assessed at 1, 24, and 48 hpi by TCID_50_ assays in MDCK cells.

### In vitro culture and infection of alveolar epithelial cells

Primary human AECs were isolated from three donors and infected^37^. In brief, AECs were infected with A(H9N2), A(H1N1pdm09), and A(H5N1) viruses at an MOI of 0.01 to study the viral replication kinetics or at an MOI of 2 to analyze the expression of cytokines (interferon-beta [IFN-β] and IFN-lambda 1 [IFN-λ1]) and chemokines (IP-10; regulated on activation, normal T-cell expressed and secreted [RANTES]; and monocyte chemoattractant protein-1 [MCP-1]). Cell lysates were collected at 24 hpi for real-time PCR studies of the expression of mRNAs encoded by the influenza matrix gene and by the genes encoding cytokines and chemokines. Mock-infected cells served as negative controls. Viral titers in supernatants were determined by TCID_50_ assays.

### Viral titration by TCID_50_ assay

MDCK cells were plated in 96-well tissue culture plates 24 h before the virus titration (TCID_50_) assay was performed. Cells were washed once with PBS and replenished with serum-free MEM supplemented with 100 U/mL penicillin, 100 µg/mL streptomycin, and 2 µg/mL of L-tosylamido 2-phenylethyl chloromethyl ketone (TPCK)-treated trypsin (Worthington, Lakewood, NJ). Serial dilutions of each virus supernatant were added to the plates in quadruplicate. The plates were observed for cytopathic effect (CPE) daily. The endpoint of the viral dilution that resulted in CPE in 50% of the inoculated wells was estimated using the Kärber method^39^.

### Real-time PCR assay

The RNA from virus-infected cells was extracted at 24 hpi by using a MiniBEST Universal RNA Extraction Kit (TaKaRa). RNA was reverse-transcribed by using oligo-dT primers with a PrimeScript™ RT Reagent Kit (TaKaRa). Expression of target gene mRNA was measured using an Applied Biosystems ViiATM 7 real-time PCR system. All procedures were performed according to the manufacturers’ instructions. The gene expression profiles of cytokines and chemokines were quantified and normalized with β-actin^40–42^.

### Mouse experiments

Groups of 6- to 8-week-old female DBA/2 J mice (Jackson Laboratory, Bar Harbor, ME) were lightly anesthetized with isoflurane and inoculated intranasally (i.n.) with 30 µL of PBS or with 30 µL of virus diluted in PBS, using 10-fold serial dilutions containing 10^1^–10^6^ EID_50_ of A/bat/Egypt/381-OP/2017 virus or A/mallard/Alberta/17/1991 virus to determine the MLD_50_ values. After virus inoculation, mice were weighed daily and monitored for mortality (actual death or loss of ≥25% of their body weight), weight loss, and any clinical signs of infection for 14 days post inoculation (dpi). To assess virus replication, groups of three mice were lightly anesthetized with isoflurane and inoculated i.n. with 10^6^ EID_50_ of A/bat/Egypt/381-OP/2017 virus or A/mallard/Alberta/17/1991 virus (in a volume of 30 μl). The mice were euthanized at 3 or 5 dpi, their nasal turbinates and lungs were collected, and the virus infectivity was titrated in eggs. All collected organs were homogenized in sterile PBS with a Qiagen Tissue Lyser II (Qiagen, Gaithersburg, MD). Organ homogenates were centrifuged at 2000 × *g* for 10 min, then the supernatants were transferred to clean tubes. Virus titers were determined by EID_50_ assays.

### Histology and immunohistochemical staining of murine tissue

The lungs and nasal turbinates of mice (*n* = 3) were collected at 3 and 5 dpi and fixed via intratracheal infusion and immersion in a 10% neutral-buffered formalin solution. Tissues were embedded in paraffin, sectioned, and stained with hematoxylin and eosin. Immunohistochemical staining was performed on serial histologic sections to determine the distribution of AIV antigens. A goat primary polyclonal antibody against influenza A/USSR/1977 (H1N1) virus (US Biological, Swampscott, MA; diluted 1:1000) was used on tissue sections that were previously subjected to antigen retrieval for 30 min at 98 °C with Dako Target Retrieval Solution, pH 9 (Agilent, Santa Clara CA). To quantify the extent of viral infection in the lungs, digital images of whole-lung sections stained for viral antigens were acquired with an Aperio ScanScope XT Slide Scanner (Leica Biosystems, Buffalo Grove, IL). Both uninfected and virus-positive regions were then manually outlined, and the areas of the outlined regions were determined with ImageScope software (Leica Biosystems).

### Pathogenicity and transmission in ferrets

Four-month-old male ferrets (Triple F Farms, Sayre, PA) that were serologically negative for influenza viruses were used in these studies. To investigate virus replication, groups of six ferrets were anesthetized and inoculated i.n. with 10^6^ EID_50_ of A/bat/Egypt/381-OP/2017 in a 1-mL volume (500 μL per nostril). Three ferrets were euthanized at each of 3 and 5 dpi, and the nasal turbinates, trachea, lung, spleen, liver, intestine, heart, and brain were collected for virus titration by EID_50_ assay.

For the transmission studies, groups of three ferrets (designated as donor ferrets) were inoculated i.n. with 10^6^ EID_50_ of test virus and then housed individually in a multilevel cage system inside an isolator. Twenty-four hours later, a naïve ferret was co-housed with an inoculated donor ferret to examine contact transmission (these were designated as direct-contact ferrets) and a naïve animal was placed in an adjacent cage, separated by a double-layered net divider, to examine respiratory droplet transmission (these were designated as airborne-contact ferrets). These cages allow the free passage of air. Nasal washes were collected from donor ferrets at 2, 4, 6, 8, 10, and 12 dpi and recipient ferrets at 2, 4, 6, 8, 10, and 12 days dpc and titrated by EID_50_ assay. Sera were collected from all animals at 14 and 21 dpi to evaluate the seroconversion.

### Replication and transmission in mallard ducks

Three donor 3–4 months old male ducks were individually infected with 5 × 10^5^ EID_50_ of A/bat/Egypt/381-OP/2017 or A/mallard/Alberta/17/1991 in 0.5 mL of PBS via the ocular, nasal, and oropharyngeal routes. Two 3-4 months old male ducks inoculated with 0.5 mL of sterile PBS were used as negative controls. To examine virus transmission, three naïve 3-4 months old male ducks (designated contact ducks) were added to each group at 2 dpi and shared the food and drinking water for 24 h before being moved to a separate cage. Body weights were determined at 3, 5, 7, 10, and 14 dpi for donor and contact ducks. Ducks were observed for clinical signs over 14 days. Oropharyngeal and cloacal swabs were collected from all birds at 2, 4, and 6 dpi to detect virus shedding. Viral titers in oropharyngeal and cloacal swabs were determined by EID_50_ assays. Blood samples were collected from donor and contact ducks at day 14 and at day 21 post infection to evaluate the seroconversion.

### Statistical analysis

Data were analyzed using two-way ANOVA with Tukey’s multiple-comparison post hoc test, and univariant log-rank analysis (survival curves) in GraphPad Prism v9.4.

### Reporting summary

Further information on research design is available in the Nature Portfolio Reporting Summary linked to this article.

## Supplementary information

Supplementary Information

Peer Review File

Reporting Summary

## Source data

Source data

## Data Availability

The authors confirm that the data supporting the findings of this study are available within the article and its Supplementary Materials. Source data are provided with this paper.
